# Copy-number analysis from genome sequencing data of 11,754 rare-disease parent-child trios: A model for identifying autosomal recessive human gene knockouts including a novel gene for autosomal recessive retinopathy

**DOI:** 10.1016/j.gimo.2024.101834

**Published:** 2024-02-29

**Authors:** Eric Olinger, Ian J. Wilson, Sarah Orr, Miguel Barroso-Gil, Ruxandra Neatu, Genomics England Research Consortium, John C. Ambrose, Prabhu Arumugam, Roel Bevers, Marta Bleda, Freya Boardman-Pretty, Christopher R. Boustred, Helen Brittain, Mark J. Caulfield, Georgia C. Chan, Greg Elgar, Tom Fowler, Adam Giess, Angela Hamblin, Shirley Henderson, Tim J.P. Hubbard, Rob Jackson, Louise J. Jones, Dalia Kasperaviciute, Melis Kayikci, Athanasios Kousathanas, Lea Lahnstein, Sarah E.A. Leigh, Ivonne U.S. Leong, Javier F. Lopez, Fiona Maleady-Crowe, Meriel McEntagart, Federico Minneci, Loukas Moutsianas, Michael Mueller, Nirupa Murugaesu, Anna C. Need, Peter O’Donovan, Chris A. Odhams, Christine Patch, Mariana Buongermino Pereira, Daniel Perez-Gil, John Pullinger, Tahrima Rahim, Augusto Rendon, Tim Rogers, Kevin Savage, Kushmita Sawant, Richard H. Scott, Afshan Siddiq, Alexander Sieghart, Samuel C. Smith, Alona Sosinsky, Alexander Stuckey, Mélanie Tanguy, Ana Lisa Taylor Tavares, Ellen R.A. Thomas, Simon R. Thompson, Arianna Tucci, Matthew J. Welland, Eleanor Williams, Katarzyna Witkowska, Suzanne M. Wood, Denize Atan, John A. Sayer

**Affiliations:** 1 Translational and Clinical Research Institute, Faculty of Medical Sciences, Newcastle University, Central Parkway, Newcastle upon Tyne, United Kingdom; 2 Center for Human Genetics, Cliniques Universitaires Saint-Luc, Brussels, Belgium; 3 Biosciences Institute, Faculty of Medical Sciences, Newcastle University, Central Parkway, Newcastle upon Tyne, United Kingdom; 4 Bristol Eye Hospital, University Hospitals Bristol & Weston NHS Foundation Trust, Bristol, United Kingdom; 5 Translational Health Sciences, Bristol Medical School, University of Bristol, Bristol, United Kingdom; 6 Renal Services, Newcastle Upon Tyne Hospitals NHS Foundation Trust, Newcastle upon Tyne, United Kingdom; 7 NIHR Newcastle Biomedical Research Centre, Newcastle upon Tyne, United Kingdom

**Keywords:** CNV analysis, Genome sequencing, NPHP1, Retinopathy, SLC66A1

## Abstract

**Purpose:**

In parent-child trios with genome sequencing data, we investigated inherited biallelic deletions to identify known and novel genetic disorders.

**Methods:**

We developed a copy-number variations analysis pipeline based on autosomal genome sequencing read depth of Genomics England 100,000 Genomes Project data from 11,754 parent-child trios and additional 18,875 non-trios. A control cohort of 15,440 cancer patients provided independent deletion frequencies.

**Results:**

Autosomal recessive (AR) modeling detected 34 distinct rare deletions that were homozygous in the proband and heterozygous in both parents. These inherited biallelic deletions were only detected in 52 trios. These “knockout” regions included 37 genes, having among them 8 with an Online Mendelian Inheritance in Man AR annotation. Deletions of *NPHP1*, followed by *OTOA*, both within segmental duplications, were the only recurrent findings explaining phenotypes in a total of 10 and 3 patients, respectively. Recurrent heterozygous *NPHP1* deletions were detected in 0.3%-0.5% of controls. We reviewed “knockout” patients for the remaining 29 genes without disease associations and identified *SLC66A1* as a likely novel cause for AR rod-cone dystrophy in 4 families.

**Conclusion:**

A tailored copy-number variations analysis of genome sequencing trio data shows that biallelic inherited gene deletions are rare, with *NPHP1* biallelic deletions causing nephronophthisis the leading finding. We propose *SLC66A1* as a novel cause for AR retinopathy.

## Graphical abstract

**Figure:**
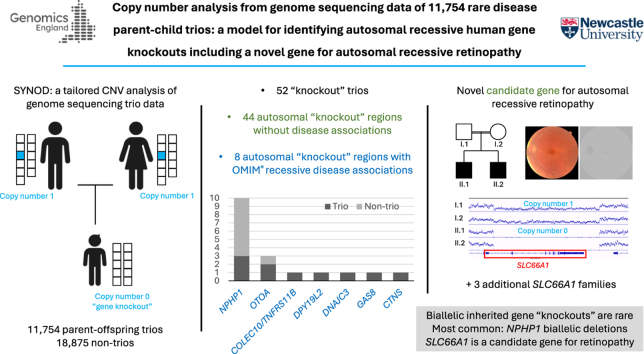

## Introduction

Genome sequencing is rapidly becoming a routine approach to investigating inherited human diseases in clinical practice.^1^ Nevertheless, data generated from genome sequencing require extensive bioinformatic analysis and filtering of variants to determine pathogenicity and remain challenging. Despite well-established workflows and software in place to process raw data,^2^ the analysis of genome sequencing data is still complex. Indeed, it is recognized that variant interpretation requires both biological and biomedical reasoning, demanding input from both scientists and clinicians, as well as bioinformatic approaches.

Copy-number variations (CNVs) are increasingly recognized to be pathogenic, especially in cases where this leads to a whole gene being deleted. Software tools to decipher CNVs across genome sequencing data sets are being utilized but are not completely accurate.

Here, we report, using Genomics England 100,000 Genomes Project (GE100kGP) data, the tailored analysis of CNV in an untargeted manner across 22 autosomes in parent-child trios and focusing on autosomal recessive (AR) inheritance to detect human “knockouts” in both known Mendelian disease-associated and non-morbid genes.

## Materials and Methods

The enrolment of study participants within the GE100kGP has previously been detailed^2^ and includes participants with rare diseases and their close relatives recruited primarily from NHS Hospitals in the United Kingdom. This included a total 30,629 probands, and among those, we selected a “patient-trio cohort” of 11,754 rare-disease families. A control cohort, not enriched for rare diseases, within the GE100kGP was identified and consisted of 15,440 individuals recruited under the cancer research domain. After informed consent, genome sequencing via the GE100kGP was performed on the probands, parents, and other family members where available. Within the rare disease cohort, a novel CNV analysis (SYNOD, https://github.com/ijwilson/SYNOD) was performed on binary alignment and map (BAM) files in 11,754 parent-child trios and then extended to 18,875 other non-trio probands. In brief, we called CNVs using a strategy based on the mean coverage depth of autosomal Ensembl 96 regions from BAM files for all rare-disease probands, parents, and control individuals. CNVs were retained after passing the following filters: (1) frequency of the deletion in the control cohort of <0.5%, (2) an inheritance pattern compatible with AR inheritance, which is a heterozygous deletion (copy number 1) in both parents and a homozygous deletion (copy number 0) in the proband, and (3) a size of at least 8000 base pairs (see Supplemental Methods for more information). The deleted genomic regions were visualized using IGV analysis of the BAM files, and we manually verified the BAM files for the known Online Mendelian Inheritance in Man (OMIM) genes, which we define as “OMIM-morbid” hits using the IGV tool. A control cohort of 15,440 cancer patients within the GE100kGP was used to determine the frequencies of heterozygous deletions. Within GE100kGP, we accessed ROH.bed files for parent-child trios and non-trios and plotted regions of homozygosity across autosomes 1 to 22. We analyzed GE100kGP data (.vcf files and clinical phenotypes) to identify additional biallelic loss-of-function (LoF) alleles in *SLC66A1* in rare-disease cases.

## Results

**Figure 1:**
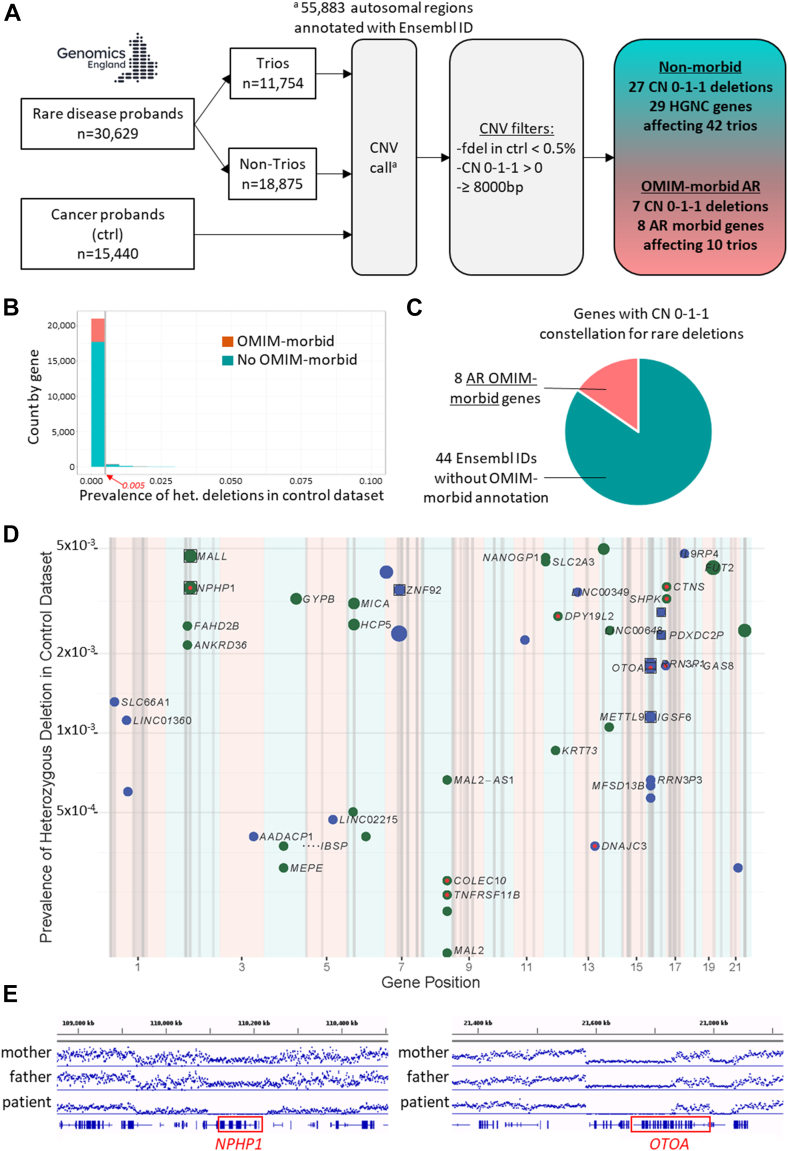
**An autosome-wide approach to CNV analysis in a rare disease cohort.** A. Flowchart of the study design. ^a^Autosomal regions annotated with Ensembl ID using Ensembl 96 in GRCh38. “fdel” denotes frequency of heterozygous deletion in the control population (cancer probands from GE100kGP). Copy number (CN) 0-1-1 denotes instances in which the proband carries a homozygous deletion (copy number 0) and both parents a heterozygous deletion (copy number 1). AR, autosomal recessive. B. Frequency plot of heterozygous deletions within OMIM-morbid and non-morbid OMIM genes. C. Pie chart showing the proportion of autosomal recessive (AR) OMIM-morbid genes among the 52 Ensembl ID autosomal regions in which rare (fdel in ctrl < 0.5%) parent-child trio CN 0-1-1 were called. D. An autosome-wide plot is shown demonstrating CN 0-1-1 parent-child trios for rare (fdel in ctrl < 0.5%) deletions. For each deletion region, HGVS gene symbols (if available) are shown. The *y*-axis is the log scale allele frequency for the corresponding heterozygous deletion (copy number 1, CN 1) in the control population. The size of the dot is proportional to the number of CN 0-1-1 parent-child trios identified and dots are colored blue for odd numbered chromosomes and green for even numbered chromosomes and pink shading is used to highlight odd numbered chromosomes. Known OMIM-morbid AR genes are colored red. Vertical gray lines represent regions of segmental duplications, and gene deletions lying within these regions are depicted by a square. E. Typical alignment charts for the 2 most prevalent CN 0-1-1 OMIM-morbid genes: *NPHP1* and *OTOA*. Average alignment data generated from BAM files using the Count application in IGVtools generating tiled data files (.tdf).

We analyzed a “patient-trio cohort” of 11,754 rare-disease families in which at least the proband and their 2 parents had genome sequencing data available. In parallel, we defined a “control cohort” of 15,440 individuals with genome sequencing data recruited under the GE100kGP GRCh38 cancer scheme (Figure 1A, Supplemental Figure 2). Using our pipeline, we called CNV in both cohorts (Figure 1A). We justified our CNV frequency filter of less than 0.5% in controls and size filter of ≥8 kb because we observed an enrichment of deletions involving OMIM-morbid genes in these rarer and larger genomic deletions (Figures 1B and C, Supplemental Figure 1).

**Figure 2:**
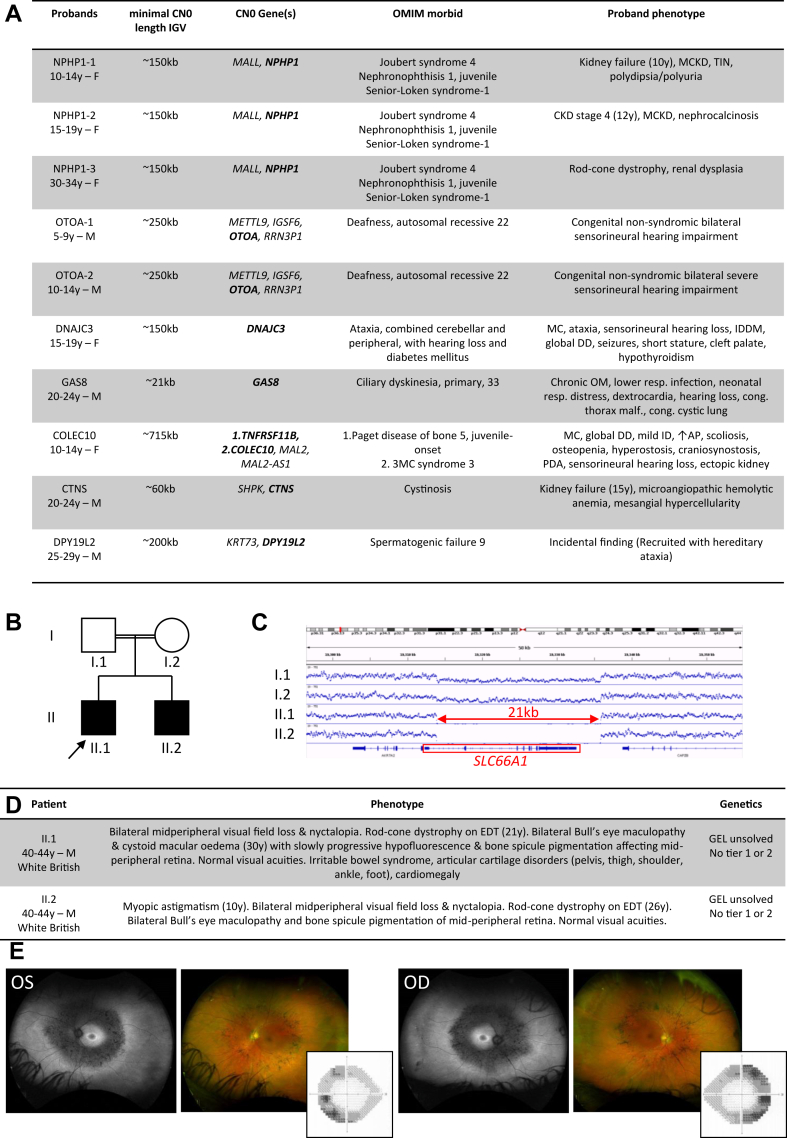
**Identified morbid and candidate gene deletions within parent-child trios and clinical phenotypes.** A. A table detailing the 10 probands from parent-child trios in which rare CN 0-1-1 (ie, instances in which the proband carries a homozygous deletion and both parents a heterozygous deletion) affecting AR OMIM-morbid genes were called. AP, alkaline phosphatase; DD, developmental delay; ID, intellectual deficiency; IDDM, insulin-dependent diabetes mellitus; MC, microcephaly; MCKD, medullary cystic kidney disease; OM, otitis media; PDA, persistent ductus arteriosus; TIN, tubulointerstitial nephritis. In bold: genes with OMIM-morbid annotation. B. Pedigree diagram of 2 siblings with retinal disease in whom homozygous deletions (CN 0) affecting *SLC66A1* were called. The parents are first cousins. C. Average alignment charts for the 2 siblings from B (II:1 and II:2) and their parents (1:1 and 1:2). The affected siblings are homozygous for the 21 kb deletion overlapping with *SLC66A1* and their parents are both heterozygous for the deletion. Average alignment data generated from BAM files using the Count application in IGVtools generating tiled data files (.tdf). D. Phenotype details for the 2 siblings (from B) homozygous for the deletion overlapping with *SLC66A1.* E. Patient II.1 developed slowly progressive adult-onset rod-cone dystrophy associated with left (OS) and right (OD) Bull’s eye maculopathy, mid-peripheral retinal hypofluorescence, and bone spicule pigmentation following the vascular arcades. Optos ultrawidefield fundal autofluorescent (black and white) images are on the left side and color images are on the right side. Inset images show corresponding visual field loss demonstrated by Humphrey 24:2 automated perimetry. See Supplemental Figures 12-14 for more phenotype details for siblings II.1 and II.2.

In the parent-child trio cohort, we detected 77 instances of copy-number-0 calls (homozygous deletion) in proband and copy-number-1 calls (heterozygous deletion) in both parents, involving a total of 52 distinct autosomal regions (Supplemental Figure 3). Taking into account that several of these calls were spanning across contiguous regions, this corresponded to a total of 34 distinct homozygous deletions (Supplemental Figure 3). Among them, inherited homozygous deletions were detected in 8 known AR OMIM-morbid genes (*NPHP1, OTOA, DNAJC3, GAS8, TNFRSF11B, COLEC10, CTNS*, and *DPY19L2*) in a total of 10 trios. The 7 distinct homozygous deletions implicating AR OMIM-morbid genes spanned from 21 kb to 750 kb (Supplemental Figures 4 and 5), and the only recurrent OMIM-morbid autosomal homozygous deletions involved *NPHP1* (3 parent-child trios, MIM ∗607100) and *OTOA* (2 parent-child trios, MIM ∗607038), whereas the other OMIM-morbid homozygous deletions were unique in the cohort (Figures 1D, E, and 2A). Eight of the autosomal copy-number-0 loci overlapped with regions known to contain segmental duplications, including the *NPHP1* and *OTOA* loci (Figure 1D), providing a potential mechanism for CNV at these loci. We manually verified all the BAM files for the OMIM-morbid hits using IGV tool (Figure 1E, Supplemental Figures 4 and 5). In total, 10 trios with homozygous deletions implicating OMIM-morbid genes have been identified, 1 of them with a homozygous contiguous gene deletion involving 2 OMIM-morbid genes (*TNFRSF11B* and *COLEC10*). In the 10 probands from the parent-child trios, the clinical phenotypes were consistent with the identified CNV and the gene(s) contained within (Figure 2A). We subsequently extended the analysis to 18,875 probands without genome sequencing data available in both parents (“non-trios”) targeting the autosomal copy-number-0 loci we had identified in the parent-child trios. Here, we detected additional patients with homozygous deletions for many of the regions, including 7 individuals homozygous for the recurrent *NPHP1* whole-gene deletion and corresponding clinical phenotypes and a single individual with the recurrent *OTOA* gene deletion and deafness but no further patients for the other identified OMIM-morbid regions (Supplemental Figures 3 and 6). In addition, we identified 1 family with an apparent de novo deletion of *NPHP1* in the proband because the mother carried no deletion for this gene, and the affected child was found to have a homozygous deletion (Supplemental Figure 7). No other de novo deletions were detected for the other morbid-OMIM loci. For the set of OMIM-morbid genes, we compared our findings with the coverage-based copy-number variant caller Canvas accessible through GE100kGP.^3^ The findings were identical except for the ∼200 kb deletion involving *DPY19L2* that was not called by Canvas.

In total, the most commonly detected homozygous deletion involved *NPHP1* followed by *OTOA*, explaining the phenotypes in 10 and 3 patients, respectively, whereas the other deletions were all unique (Supplemental Figure 8). In contrast to *OTOA*, in which genome-wide analysis identified large runs of autozygosity in 2 out of 3 probands, evidence for autozygosity by descent was only detected in 3 out of 10 individuals with homozygous *NPHP1* deletions (Supplemental Figures 9 and 10), suggesting that that heterozygous *NPHP1* deletions are recurrent in the general population and may arise commonly de novo. As a proof of principle, we also applied our CNV calling pipeline (https://github.com/ijwilson/SYNOD) to genome sequencing data from the 1000 Genomes Project. Targeting the *NPHP1* locus, out of a total of 2600 individuals, 14 individuals with various ethnic backgrounds were identified with a heterozygous *NPHP1* deletion, suggesting a similar prevalence compared with our GE100kGP control cohort (0.54% vs 0.35%) (Supplemental Figure 11).

Finally, we reviewed the phenotypes for individuals homozygous for inherited deletions in genes not previously implicated in human disease (Figure 1D, Supplemental Figure 3). Among the 29 genes with HUGO Gene Nomenclature Committee (HGNC) symbols, *SLC66A1* was an interesting human disease candidate gene. The identified proband had an affected sibling and both were homozygous for an inherited 21 kb deletion (and their consanguineous parents were both heterozygous for the deletion) spanning nearly the entirety of *SLC66A1* (Figure 2B and C). Both siblings presented with slowly progressive adult-onset rod-cone dystrophy associated with Bull’s eye maculopathy and mid-peripheral visual field loss and were labeled unsolved by the Genomics England diagnostics pipeline (see Figure 2D and E, Supplemental Figures 12-14). Before recruitment to GE100kGP, both siblings had been tested for pathogenic variants in *RPE65* and had further genetic tests as participants of a research study called the UK Inherited Retinal Dystrophy Consortium Retinits Pigmentosa Genome Project. *SLC66A1* has been suggested as a candidate gene for inherited retinal disease.^4^ We then screened the GE100kGP rare disease data set for Indels and SNVs in this gene and identified 3 further patients with homozygous predicted LoF variants in *SLC66A1* (1 nonsense and 2 frameshifting, which were verified on the BAM file data). Importantly, all 3 of them presented with progressive rod-cone dystrophy, although 1 of the patients was recruited under “early-onset severe rod-cone dystrophy.” In the latter case, an additional homozygous nonsense variant was identified in *SPATA7* (autosomal recessive retinitis pigmentosa type 94 and Leber congenital amaurosis type 3, MIM #604232), suggesting that this individual could have a more severe digenic retinal disease. The other 2 cases were unsolved after routine evaluation by the Genomics England diagnostics team, and no further tier 1 or tier 2 genetic variants (suggesting pathogenicity) were reported (Supplemental Figure 15). There are no individuals homozygous for predicted *SLC66A1* LoF single-nucleotide variants or indels in gnomAD v4.0.0. Only 1 non-intronic 8.16 kb deletion is described (removing the C-terminal exons 3-8 of *SLC66A1*) in gnomAD. This deletion is described in a heterozygous state in 1 European individual. Similarly, there are no reported homozygous deletions involving *SLC66A1* in Decipher, ClinVar, or Database of Genomic Variants. In summary, based on 2 siblings with rod-cone dystrophy with homozygous *SLC66A1* whole-gene deletions and further 3 rod-cone dystrophy cases with homozygous predicted LoF variants in *SLC66A1*, we suggest *SLC66A1* may be a novel gene for AR adult-onset rod-cone dystrophy.

## Discussion

Studying genetic LoF variants in large human populations is extremely informative and has allowed significant progress in understanding genotype-phenotype correlations.^5^, ^6^, ^7^ Homozygous whole-gene deletions are the best example of an unambiguous LoF allele; yet, their detection within large population data sets such as GE100kGP remains incomplete.^1^^,^^2^^,^^8^ There are a variety of tools that can be applied to the analysis of CNV within genome sequencing data sets; however, these tools are still evolving.^9^ Here, we used a novel analysis of genomic wide CNVs, specifically looking for inherited autosomal homozygous deletions. This allowed accurate detection of genomic regions containing annotated genes, many of which overlapped with regions of segmental duplications. Then, using a region-targeted approach, we were able to identify single probands with homozygous deletions, validating the parent-child trio approach for the detection of homozygous deletions. The findings presented here show that whole-gene deletions in OMIM-morbid genes are rare, but an adapted CNV analysis does allow their detection allowing cases to be genetically solved. Indeed, based on our AR CNV model, we provided a molecular diagnosis in 10 out of 11,754 parent-child trios and in 8 out of 18,875 non-trios in the targeted follow-up analysis. Except for 1 *NPHP1* trio, all these patient trios did not have a diagnosis after routine evaluation by the Genomics England diagnostics pipeline.

Using our tailored pipeline and AR filtering, *NPHP1* whole-gene deletions were the most frequent homozygous deletions within the GE100kGP rare-disease cohort we analyzed. Indeed, when considering only rare-disease probands with advanced kidney disease (recruited in GE100kGP under the keywords “end-stage kidney disease,” chronic kidney disease stage 4,” and “chronic kidney disease stage 5”), the yield of homozygous deletions in *NPHP1* is ∼1.2% (11 homozygous *NPHP1* deletions out of 959 probands with advanced kidney disease). These deletions, which typically involve part of the neighboring gene *MALL*, are located near a region of segmental duplication.^10^ Biallelic pathogenic variants in *NPHP1* are the most common cause of infantile nephronophthisis,^11^, ^12^, ^13^, ^14^ and homozygous whole-gene deletions typically account for 25% of all *NPHP1* pathogenic variants.^13^ Recent research has also identified *NPHP1* deletions in adult patients with unexplained kidney failure^15^ suggesting that these patients may often be clinically and genetically underdiagnosed. Indeed, within this adult kidney failure cohort, the prevalence of *NPHP1* whole-gene deletions was estimated to be 0.5%, slightly lower than when compared with our estimated prevalence in a mostly pediatric cohort.^15^ The fact that *NPHP1* lies in a segmental duplication region suggests that it may be susceptible to de novo gene deletions and that the finding of homozygous *NPHP1* deletions does not imply parental consanguinity. Our data showed examples of both a de novo deletion in *NPHP1* and a lack of autozygosity by descent in the majority of *NPHP1* deletion parent-child trios. The clinical phenotypes in our *NPHP1* deletion probands were consistent with those expected, including kidney failure secondary to nephronophthisis and retinal dystrophy, the combination of which is called Senior-Løken syndrome.^16^ *NPHP1* deletions are also a rare cause of Joubert syndrome related disorder,^17^ but we did not find cases with this precise phenotype. We were also able to confirm that the carrier frequency of *NPHP1* heterozygous deletions was 0.35% within a non-rare-disease cohort (the GE100kGP cancer cohort) and 0.54% within the 1000 Genomes project.

Although there are examples of biallelic LoF variants being tolerated in human gene variation,^5^ their identification may be used to prioritize candidate genes for new human diseases. Here, we identified that a homozygous deletion affected *SLC66A1* in 2 siblings with retinal dystrophy and further evidence that this may be a novel disease-causing gene in 3 other families with homozygous LoF alleles (small frameshifting deletions) in this gene with similar retinal phenotypes. Three patients with AR retinitis pigmentosa from 2 consanguineous families have previously been identified with homozygous missense (predicted to affect protein function) or frameshifting variants in *SLC66A1*, suggesting LoF as the disease mechanism.^4^ The previously reported case was clinically comparable, including adult-onset disease, progressively reduced full field electroretinogram responses, peripheral visual field loss, and bone spicule-like pigmentary changes, although detailed analysis revealed some differences^4^ (see Supplemental Clinical Information for full description and comparison). *SLC66A1* is expressed in multiple human retinal cells, including the photoreceptors.^4^ There is a growing list of OMIM-morbid solute carrier (SLC) genes that include a diverse spectrum of human diseases, including neurological, metabolic, and retinal diseases.^18^ Previous studies have established the encoded SLC66A1 protein as a mammalian lysosomal cationic amino acid exporter, related to the cystinosin transporter family.^19^ Future studies deciphering the role of *SLC66A1* in the retina, as well as the functional consequences of absent SLC66A1 in patient cells and model systems are needed to definitively establish the gene-disease association,^20^ although the 4 families presented here and the 2 families described previously^4^ strongly suggest a causal relationship with inherited retinal disease.

Our study provides a robust approach to identifying homozygous deletions affecting annotated genes using genome sequencing data, but it has some limitations. Our GE100kGP cohort was largely white British and was enriched for rare diseases. We restricted our genome sequencing read depth analysis to homozygous deletions that were inherited from heterozygous parents and affecting annotated genes in autosomes; therefore, deletions in X-linked genes and de novo CNVs have not been systematically analyzed within this study. To increase specificity of the copy-number-0 calls, our initial approach relied on using parent-patient trios to reliably identify rare homozygous deletions that were inherited from both parents and only allowed a targeted approach to singlets. Genome sequencing allowed better characterization of CNV using a variety of computational approaches. The combination of several tools with complementary strength and limitations is likely the provide the best yield in terms of CNV detection.^21^ SYNOD has performed well for regions with segmental duplications at both or either end, where methods that rely on identifying break points have problems with alignment and can be added to the toolbox for straightforward “whole-gene” CNV screening of large genome sequencing databases.

## Conclusions

Homozygous gene deletions within the human genome remain exceedingly rare events and are not readily detected by routine genome sequencing analysis pipelines. Here, we demonstrate that an untargeted approach in parent-patient trios and singlets is valuable in identifying patients with AR disease and provides human gene “knockouts” for the evaluation of candidate genes. In this manner, we have determined *SLC66A1* as a strong candidate gene for inherited retinal disease by identifying 2 siblings with whole-gene deletions of *SLC66A1* and an additional 3 cases with homozygous LoF variants in this gene and similar retinal phenotypes. Further work and validation is needed to determine how best to incorporate CNV analysis into clinical practice and to investigate the biological and clinical significance of detected whole-gene deletions that are not already associated with human disease genes.

## Data Availability

Data that support the findings of this study are available on request from the corresponding author with patient privacy restrictions. Script files and workflow for CNV calling pipeline SYNOD (Simple copY Numbers frOm Depth) have been deposited under https://github.com/ijwilson/SYNOD.

## Conflict of Interest

The authors declare no conflicts of interest.

## Additional Information

Supplementary methods Supplementary acknowledgements Supplementary clinical information and comparison with SLC66A1 cases reported by Millo et al. Supplementary results (Figures S1-S15)
