# Supplementary material for: Copy-number analysis from genome sequencing data of 11,754 rare-disease parent-child trios: A model for identifying autosomal recessive human gene knockouts including a novel gene for autosomal recessive retinopathy
Source: Genet Med Open. 2024 Feb 29;2:101834. doi: 10.1016/j.gimo.2024.101834 (PMC11613694; doi:10.1016/j.gimo.2024.101834)
Supplement: Supplementary methods — Supplementary acknowledgements Supplementary clinical information and comparison with SLC66A1 cases reported by Millo et al. Supplementary results (Figures S1-S15) [file mmc1.pdf]

Supplementary data

Copy number analysis from genome sequencing data of 11,754 rare disease parent-child trios: a model for identifying autosomal recessive human gene knockouts including a novel gene for autosomal recessive retinopathy

Eric Olinger, Ian J. Wilson, Sarah Orr, Miguel Barroso-Gil, Ruxandra Neatu, Genomics England Research Consortium, Denize Atan and John A. Sayer

Supplementary methods

Calling copy number variation

Participant data

Within the Genomics England 100,000 Genomes Project (GE100kGP), genome sequencing data were determined by rare disease proband individuals assembled into GRCh38. These were performed using "rare disease" individuals in the main program under Labkey database version 14. Those individuals where genome sequencing data was available from both parents were collected into 11,754 parent-child trios. In addition, there were 18,875 non-trio probands (i.e., genome sequencing data not available for both parents). There were no filtering steps applied for solved individuals or for relatives. A control cohort was taken from germline sequenced genomes from individuals within the GE100kGP cancer patient set using Labkey version 15.

Regions

The defined genomic regions were Ensembl genes sets for human version 96 for GRCh38. This was available within GE100kGP, here called "E96R". The same regions can be downloaded from https://ftp.ensembl.org/pub/release96/gtf/homo_sapiens/Homo_sapiens.GRCh38.96.gtf.gz. Using Ensembl ID annotations for genes across all autosomes and an 8kb window we identified 23,443 autosomal gene regions, the "deletion test set". The 8kb size threshold applied to the E96R gene set was chosen to retain disease genes and disease gene candidates (protein coding or known OMIM morbid genes) while filtering out "junk" such as pseudogenes or non-coding RNA elements and to be large enough to provide reliable mean depth data (Figure S1).

OMIM

OMIM annotation of genes was taken from the file genemap2.txt generated from OMIM.org on 09/09/2022.

Segmental duplication

Regions of segmental duplication (also known as low copy repeats) were downloaded from the UCSC Segmental Duplication track on UCSC for genome build GRCh38. Regions between pairs of sequences from the same chromosome with sequence similarity > 99% and that overlapped at least one of the “deletion test set” were selected. This gave 108 regions after removing duplicates and combining overlapping regions.

Calling CNVs

The program sambamba using the depth region option was used to extract the mean depth on all autosomal gene regions of “E96R” from BAM files for all probands, parents (parent-child trios and non-trio probands) and control individuals, to get mean read counts for each region. For each individual, each genomic region count is divided by the median read count over all genomic regions allowing the approximate copy number per region to be generated. Integer copy numbers 0,1,2,3,4 can be called from $r<0.4$, $0.4 \le r<1.5$, $1.5 \le r<2.5$, $2.5 \le \ge r<3.5$, and $r \ge 3.5$.

The method SYNOD (Simple copY Numbers frOm Depth) is described fully on the github https://github.com/ijwilson/SYNOD which includes instructions for repeating the GE100kGP analysis and also analysing 1000 Genomes data.

Controls

The control cohort of individuals was analysed for copy number 1 regions and was used to obtain OMIM-morbid and other genes that had a low (<0.5%) frequency of deletions.

Parent-child trios and non-trio probands

The CNV calls in parent-child trios were collected into trios and regions where parents both had a deletion on one chromosome (heterozygous deletion - copy number 1) were used to investigate homozygous deletions (copy number 0) in the proband. Regions identified in this manner were then investigated in a non-trio i.e. singlet cohort looking specifically for homozygous deletion (copy number 0).

Supplementary acknowledgements

The Genomics England 100,000 Genomes Project analysis and ethics statement

The Genomics England 100,000 Genomes Project is a consortium of genomic sequences from NHS rare disease or cancer patients, to enhance genetic diagnosis, and better healthcare research. All participants of the 100,000 Genomes Project have provided written consent to access their anonymized genetic and clinical data for research purposes. The Genomics England 100,000 Genomes Project was approved by the Health Research Authority Research Ethics Committee East of England – Cambridge South (REC Ref 14/EE/1112).

Supplementary clinical information and comparison with *SLC66A1* cases reported by Millo et al (PMID 35486108).

Both siblings with *SLC66A1* homozygous deletions reported in this study presented in their 20s with visual field loss that was detected incidentally during routine eye tests at their local optician practices. On further questioning, they also had nyctalopia (night blindness). Central vision and visual acuities were normal for both siblings throughout their duration of follow-up until their 40s (present date). Both siblings had mid-peripheral visual field loss associated with bone spicule pigmentation and hypofluorescence limited to the mid-peripheral retina, just outside the retinal vascular arcades, with sparing of the far retinal periphery. There isn't any particular sparing of the temporal periphery. The optics discs and retinal vessels appear normal. The sibling with the more severe phenotype had unrecordable rod and cone responses in their full field electroretinogram (ERG) at presentation and developed cystic changes in the central macula region during their follow-up that had no effect on their visual acuity. The visual electrophysiology of the second sibling showed their rod > cone responses were smaller and more delayed. Neither sibling had electrooculograms (EOGs).

The Millo et al. (PMID: 35486108) report only provides clinical details for one case in the consanguineous Kurdish-Jewish family. The case was diagnosed slightly later (in their 30s) after presenting with night blindness. The case had very severe peripheral visual field loss that only spared the central 5 degrees of visual field (aka tunnel vision) whereas our cases had sparing of their far peripheral visual field, outside the central 30 degrees, which is why their field loss looks doughnut shaped with Goldmann perimetry. In Millo et al. the bone spicule pigmentation does appear to extend further beyond the retinal vascular arcades to the far peripheral retina with quite extensive associated atrophy of the retinal pigment epithelium/retina; these retinal changes match the pattern of visual field loss. In contrast, the bone spicule pigmentation in our cases is more subtle and limited to just outside the vascular arcades, sparing the far peripheral retina; this is why the field loss in our cases is doughnut shaped rather than tunnel shaped. They report their patient had optic disc pallor and attenuated retinal vessels: both these signs commonly occur in longstanding RP. The patient in the Millo et al. report was followed up until age 72

and the images may have been taken at that age (rather than on presentation), therefore it is possible that our cases will also lose their far peripheral visual field, develop optic disc pallor and attenuated retinal blood vessels when they reach their 70s. There is some evidence of this from the slow progression of their visual field loss over time.

The Millo et al. study has also provided cross-sectional OCT images of the macula and far peripheral retina of their case, showing severe thinning of the photoreceptor and outer nuclear layers of the retina. This is the expected consequence of rod photoreceptor death caused by any type of rod-cone dystrophy or RP. Their case does not appear to have any cystoid macular changes but the morphology of the macula in both eyes is not entirely normal, which is probably why the visual acuity of their case is slightly reduced (whereas the visual acuity of our cases is normal, despite the cystoid macular changes in one of them). They also report that the foveal zone of their case was hyperfluorescent whereas the opposite was true of our cases who had central hypofluorescence surrounded by a ring of hyperfluorescence – the classic Bull's eye appearance on fundus autofluorescence.

In Millo et al., Family 2 was a consanguineous Bedouin family with 4 siblings affected with autosomal recessive retinitis pigmentosa but without further clinical information.

Supplementary results

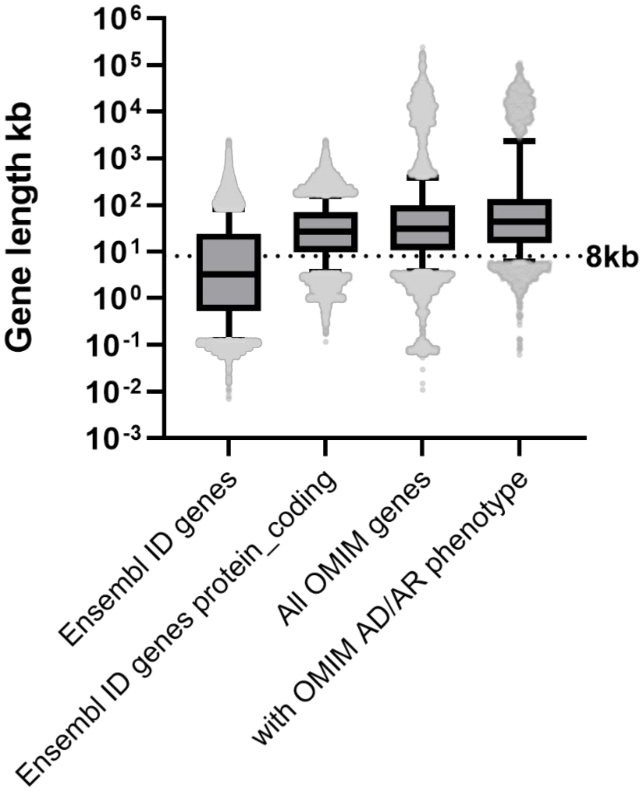

Figure S1. Relationship between gene annotation and gene length

Ensembl ID genes contains the entire Ensembl genes sets for human version 96 for GRCh38 (n= 58,731). Ensembl ID genes protein coding is the biotype "protein coding" subset of the former (n=19,937). All OMIM genes (n=17,099) and genes with OMIM autosomal dominant or recessive phenotypes (n=4,627) are those genes annotated with a gene and phenotype MIM number, respectively. Box and whiskers depict percentiles 10, 25, 50, 75, 90. Dotted line shows threshold chosen to test for "whole gene knock-outs" in our dataset.

	Parent-child trios	Single patients (non-trios)	Controls (cancer cohort)
Numbers	11,754	18,875	15,440
Self-reported ethnic category	White (British & non-British): 8051 (68.5%)<br>Asian or Asian British: 1297 (11.0%)<br>Black or Black British: 181 (1.5%)<br>Mixed background: 416 (3.5%)<br>Any other background: 171 (1.5%)<br>Not stated: 1638 (14.0%)	White (British & non-British): 13059 (69.2%)<br>Asian or Asian British: 1351 (7.1%)<br>Black or Black British: 580 (3.1%)<br>Mixed background: 419 (2.2%)<br>Any other background: 335 (1.8%)<br>Not stated: 3131 (16.6%)	White (British & non-British): 11115 (72.0%)<br>Asian or Asian British: 498 (3.2%)<br>Black or Black British: 376 (2.4%)<br>Mixed background: 110 (0.7%)<br>Any other background: 181 (1.2%)<br>Not stated: 3160 (20.5%)
Age	7y (3y, 14y)	42y (22y, 56y)	65y (54y, 73y)
Gender	Female: 43.3%	Female: 51.1%	Female: 56.2%

Figure S2. Cohort summary data

Summary of cohorts derived from the GE100kGP including parent-child-trios, singlet patients (non-trios) from the rare disease dataset and control cohort derived from the cancer cohort. Age: median, 1st and 3rd quartiles. The ethnic categories are self-reported within Genomics England and are to be regarded as ethnic groupings that also consider historical and cultural backgrounds, as opposed to racial groupings based only on physical traits.

chr	ensembl ID	gene symbol	CN 0-1-1 (n)	CN 0 non-trios (n)	OMIM-morbid
1	ENSG00000040487	*SLC66A1*	1	0	N
1	ENSG00000233973	*LINC01360*	1	1	N
1	ENSG00000285462	*NO HGNC SYMBOL*	1	0	N
2	ENSG00000144199	*FAHD2B*	1	3	N
2	ENSG00000135976	*ANKRD36*			N
2	ENSG00000144063	*MALL*	**3**	**7**	N
**2**	**ENSG00000144061**	***NPHP1***			**Y**
3	ENSG00000240602	*AADACP1*	1	0	N
4	ENSG00000029559	*IBSP*	1	0	N
4	ENSG00000152595	*MEPE*			N
4	ENSG00000250361	*GYPB*	2	5	N[1]
5	ENSG00000249128	*LINC02215*	1	0	N
6	ENSG00000281706	*NO HGNC SYMBOL*	1	1	N
6	ENSG00000284528	*MICA*	2	0	N
6	ENSG00000286337	*HCP5*			N
6	ENSG00000220291	*NO HGNC SYMBOL*	1	0	N
7	ENSG00000231704	*NO HGNC SYMBOL*	3	9	N
7	ENSG00000244550	*NO HGNC SYMBOL*	5	2	N
7	ENSG00000282381	*NO HGNC SYMBOL*	2	0	N
7	ENSG00000146757	*ZNF92*			N
**8**	**ENSG00000164761**	***TNFRSF11B***	**1**	**0**	**Y**
**8**	**ENSG00000184374**	***COLEC10***			**Y**
8	ENSG00000147676	*MAL2*			N
8	ENSG00000253972	*MAL2-AS1*			N
8	ENSG00000253398	*NO HGNC SYMBOL*			N
11	ENSG00000214883	*NO HGNC SYMBOL*	1	1	N
12	ENSG00000176654	*NANOGP1*	1	0	N
12	ENSG00000059804	*SLC2A3*			N
12	ENSG00000186049	*KRT73*	1	0	N
**12**	**ENSG00000177990**	***DPY19L2***	**1**	**0**	**Y**
12	ENSG00000255583	*NO HGNC SYMBOL*			N
13	ENSG00000231238	*LINC00349*	1	0	N
**13**	**ENSG00000102580**	***DNAJC3***	**1**	**0**	**Y**
14	ENSG00000258772	*NO HGNC SYMBOL*	2	9	N
14	ENSG00000257900	*NO HGNC SYMBOL*	1	0	N
14	ENSG00000259129	*LINC00648*	1	0	N
16	ENSG00000197006	*METTL9*	**2**	**1**	N
16	ENSG00000140749	*IGFS6*			N
**16**	**ENSG00000155719**	***OTOA***			**Y**
16	ENSG00000248124	*RRN3P1*			N
16	ENSG00000140743	*NO HGNC SYMBOL*	1	0	N
16	ENSG00000230872	*MFSD13B*			N
16	ENSG00000257122	*RRN3P3*			N
16	ENSG00000196696	*NO HGNC SYMBOL*	1	0	N
16	ENSG00000255185	*PDXDC2P*			N
**16**	**ENSG00000141013**	***GAS8***	**1**	**0**	**Y**
17	ENSG00000197417	*SHPK*	**1**	**0**	N[2]
**17**	**ENSG00000040531**	***CTNS***			**Y**
18	ENSG00000262081	*IL9RP4*	1	1	N
19	ENSG00000176920	*FUT2*	4	6	N[3]
21	ENSG00000224790	*NO HGNC SYMBOL*	1	0	N
22	ENSG00000236540	*NO HGNC SYMBOL*	3	0	N

Figure S3. Identified autosomal regions with homozygous deletions (copy number 0, CN 0) in parent-child trios and non-trios

Table showing all the 52 Ensembl ID regions affected by CN 0-1-1 calls (i.e., instances where the proband carries a homozygous deletion and both parents a heterozygous deletion) in parent-child trios and the numbers of corresponding homozygous deletion (CN 0) calls in non-trios (singletons or only 1 parent recruited with genome sequencing data available). Grey shadings highlight contiguous regions affected by the same deletion. Bold type represents genes with autosomal recessive OMIM-morbid annotation. [1][Blood group, Ss], MIM#111740; {Malaria, resistance to}, MIM#611162, [2][Sedoheptulokinase deficiency], MIM#617213; [3][Bombay phenotype, digenic], MIM#616754; {Vitamin B12 plasma level QTL1}, MIM#612542

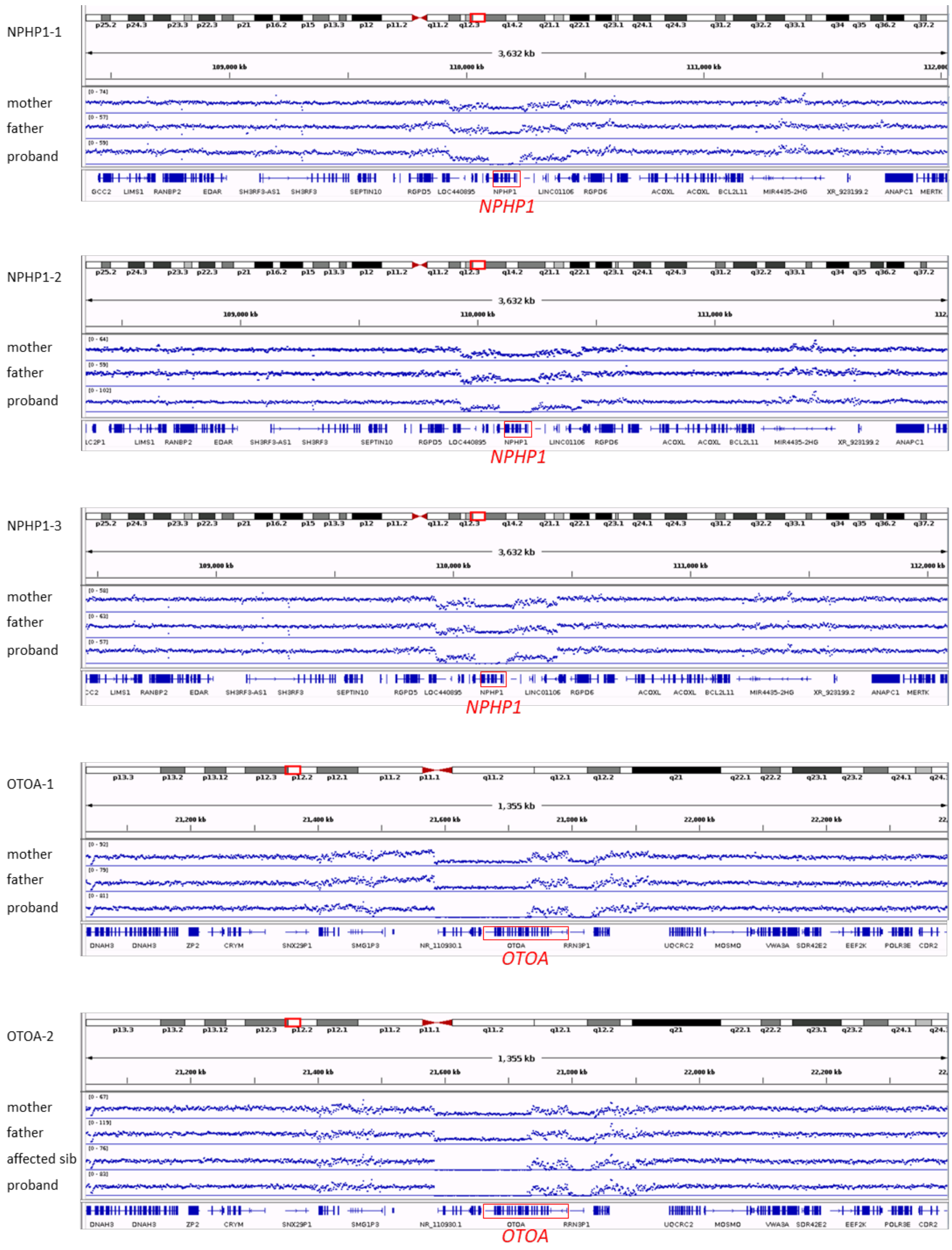

Figure S4. Average alignment charts for parent-child trios with *NPHP1* or *OTOA* deletions

Average alignment charts in parent-child trios for the homozygous deletions affecting *NPHP1* and *OTOA*. Average alignment data were generated from BAM files using the Count application in IGVtools generating tiled data files (.tdf).

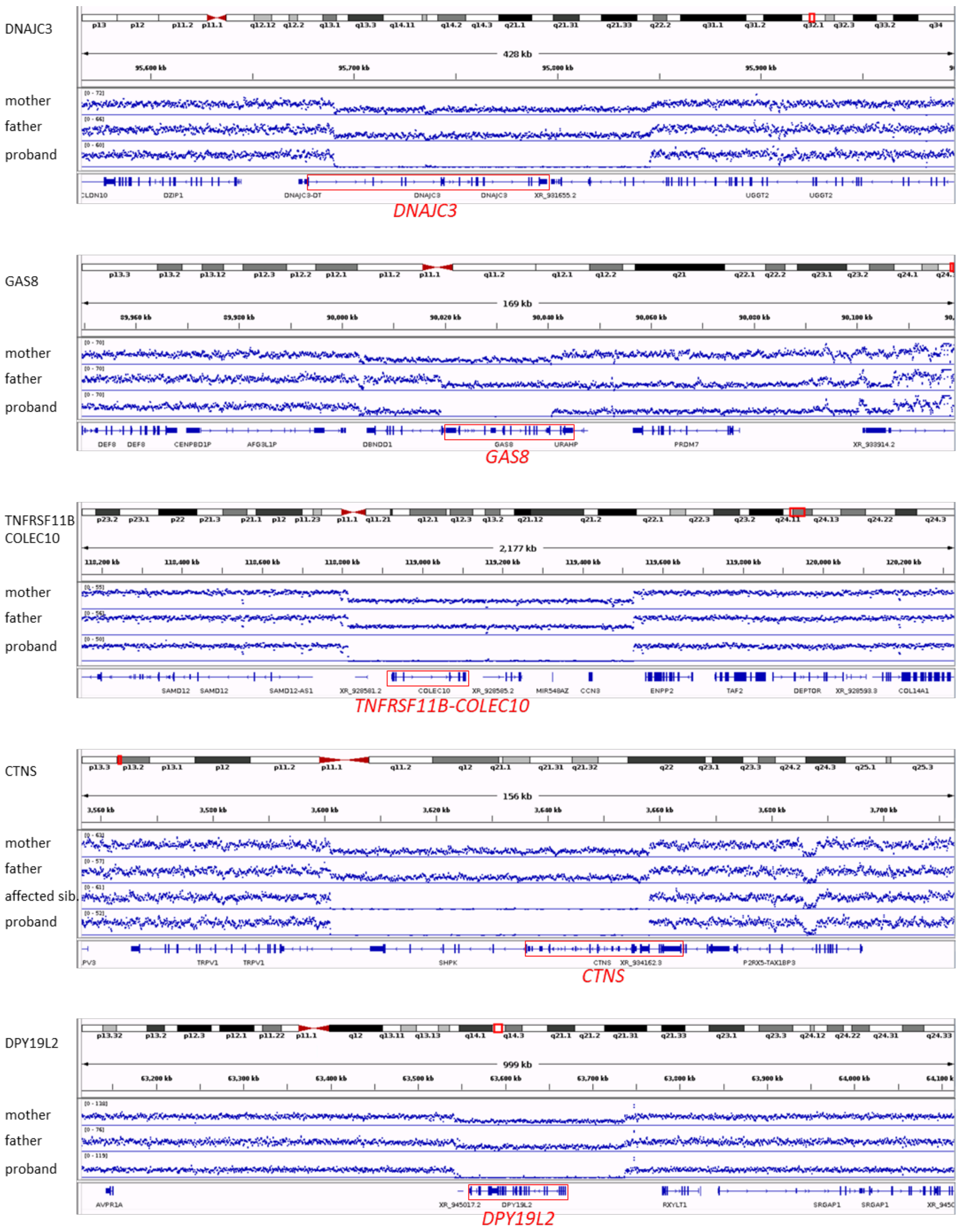

Figure S5. Average alignment charts for parent-child trios with deletions affecting other OMIM-morbid genes

Average alignment charts in trios for deletions affecting autosomal recessive OMIM-morbid genes. Average alignment data generated from BAM files using the Count application in IGVtools generating tiled data files (.tdf).

Probands	minimal CN0 length IGV	CN0 Gene(s)	OMIM morbid	Proband phenotype
NPHP1-1<br>45-49y - M	~150kb	*MALL*, ***NPHP1***	Joubert syndrome 4<br>Nephronophthisis 1, juvenile<br>Senior-Loken syndrome-1	Kidney failure (23y), aHT, hypopituitarism (34y), aortic valve insufficiency
NPHP1-2<br>50-54y - M	~150kb	*MALL*, ***NPHP1***	Joubert syndrome 4<br>Nephronophthisis 1, juvenile<br>Senior-Loken syndrome-1	Kidney failure (33y), rod-cone dystrophy, aHT, T1DM, diaphragmatic hernia, diverticular disease, combined kidney-pancreas Tx
NPHP1-3<br>45-49y - M	~150kb	*MALL*, ***NPHP1***	Joubert syndrome 4<br>Nephronophthisis 1, juvenile<br>Senior-Loken syndrome-1	Kidney failure (28y), TIN, retinal dystrophy, aHT
NPHP1-4<br>25-29y - F	~150kb	*MALL*, ***NPHP1***	Joubert syndrome 4<br>Nephronophthisis 1, juvenile<br>Senior-Loken syndrome-1	Kidney failure (22y), TIN, dextrocardia, abdominal situs inversus, chronic sinusitis, cough, recruited under PCD, DD (dyslexia and alexia), aHT, hearing loss
NPHP1-5<br>20-24y - M	~150kb	*MALL*, ***NPHP1***	Joubert syndrome 4<br>Nephronophthisis 1, juvenile<br>Senior-Loken syndrome-1	Unexplained kidney failure, aHT
NPHP1-6<br>55-59y - M	~150kb	*MALL*, ***NPHP1***	Joubert syndrome 4<br>Nephronophthisis 1, juvenile<br>Senior-Loken syndrome-1	Kidney failure (33y), gout (33y), aHT, bronchiectasis, cough, sensorineural hearing loss
NPHP1-7<br>20-24y - M	~150kb	*MALL*, ***NPHP1***	Joubert syndrome 4<br>Nephronophthisis 1, juvenile<br>Senior-Loken syndrome-1	CAKUT – renal dysplasia
OTOA-1<br>15-19y - M	~250kb	*METTL9*, *IGSF6*, ***OTOA***, *RRN3P1*	Deafness, autosomal recessive 22	Congenital profound bilateral non-syndromic sensorineural hearing impairment

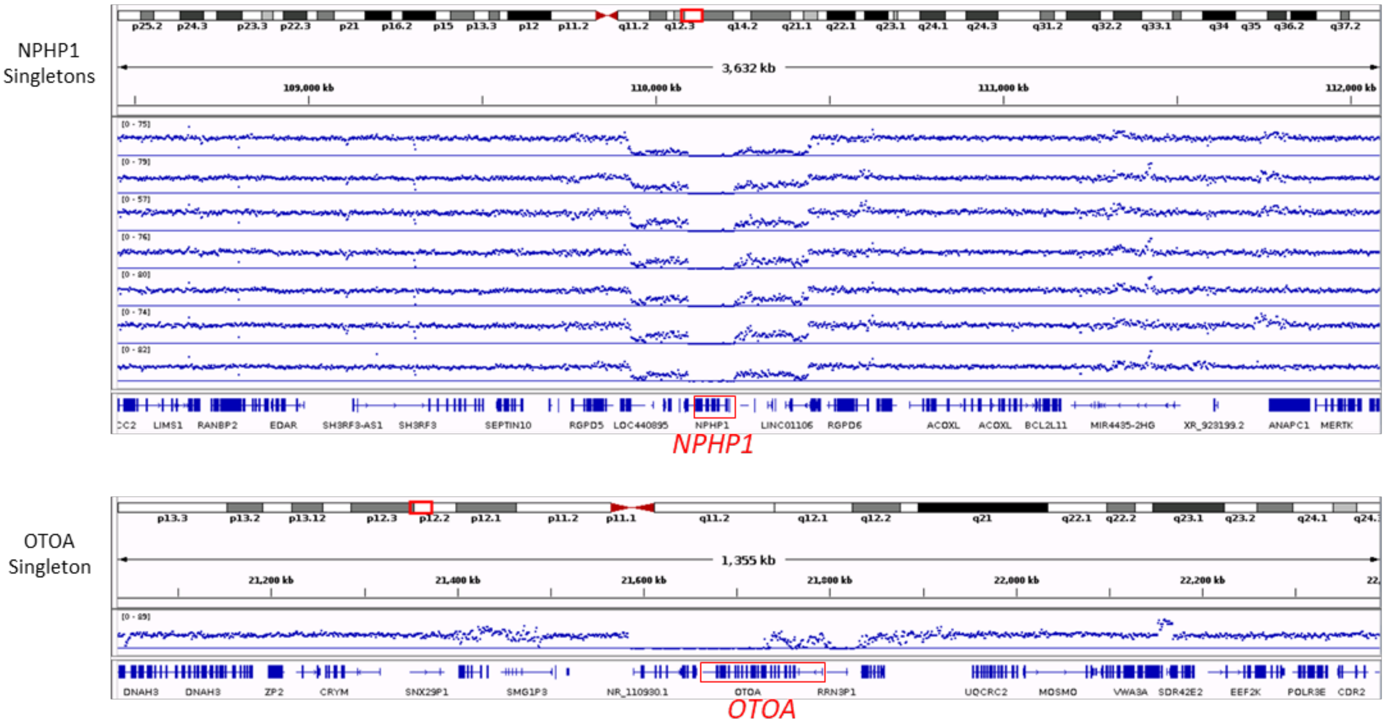

Figure S6. *NPHP1* and *OTOA* deletions and clinical phenotypes in non-trio patients

Phenotype table and average alignment charts for non-trios (singletons or only 1 parent recruited) with homozygous deletions involving *NPHP1* and *OTOA*. CN0 denotes homozygous deletions (copy number 0).

aHT, arterial hypertension; DD, developmental delay; PCD, primary cilia dyskinesia; T1DM, type 1 diabetes mellitus; TIN, tubulointerstitial nephritis; Tx, transplantation.

In bold: genes with OMIM-morbid annotation.

Bottom: Average alignment data generated from BAM files using the Count application in IGVtools generating tiled data files (.tdf).

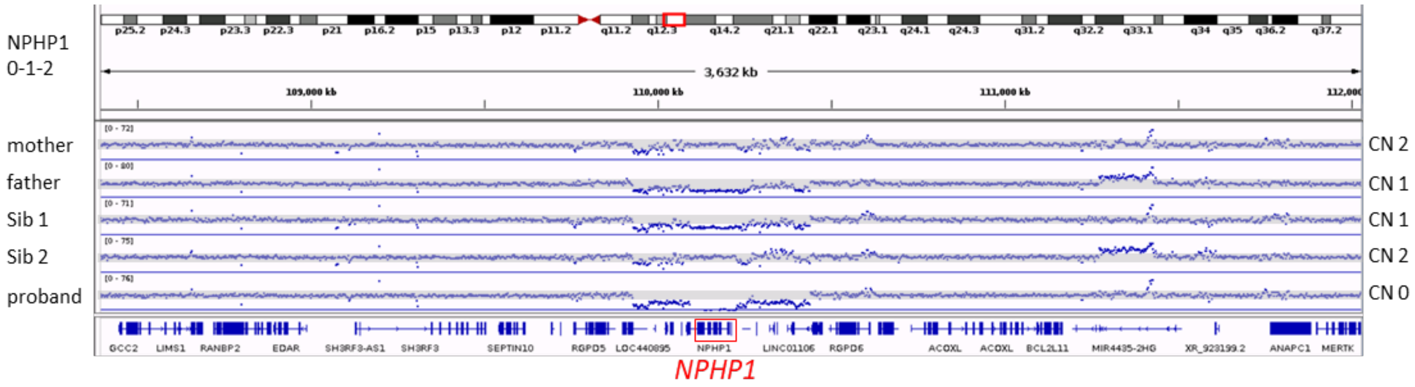

Proband	minimal CN0 length IGV	CN0 Gene(s)	OMIM morbid	Proband phenotype
NPHP1<br>20-24y – F	~150kb	*MALL*, ***NPHP1***	Joubert syndrome 4<br>Nephronophthisis 1, juvenile<br>Senior-Loken syndrome-1	Kidney failure (13y), kidney cysts, TIN, NPHP, aHT

Figure S7. Average alignment charts and phenotype information for family with a *de novo NPHP1* deletion

Average alignment data generated from BAM files using the Count application in IGVtools generating tiled data files (.tdf). aHT, arterial hypertension; NPHP, nephronophthisis; TIN, tubulointerstitial nephritis. In bold: genes with OMIM-morbid annotation. CN0 denotes homozygous deletions (copy number 0).

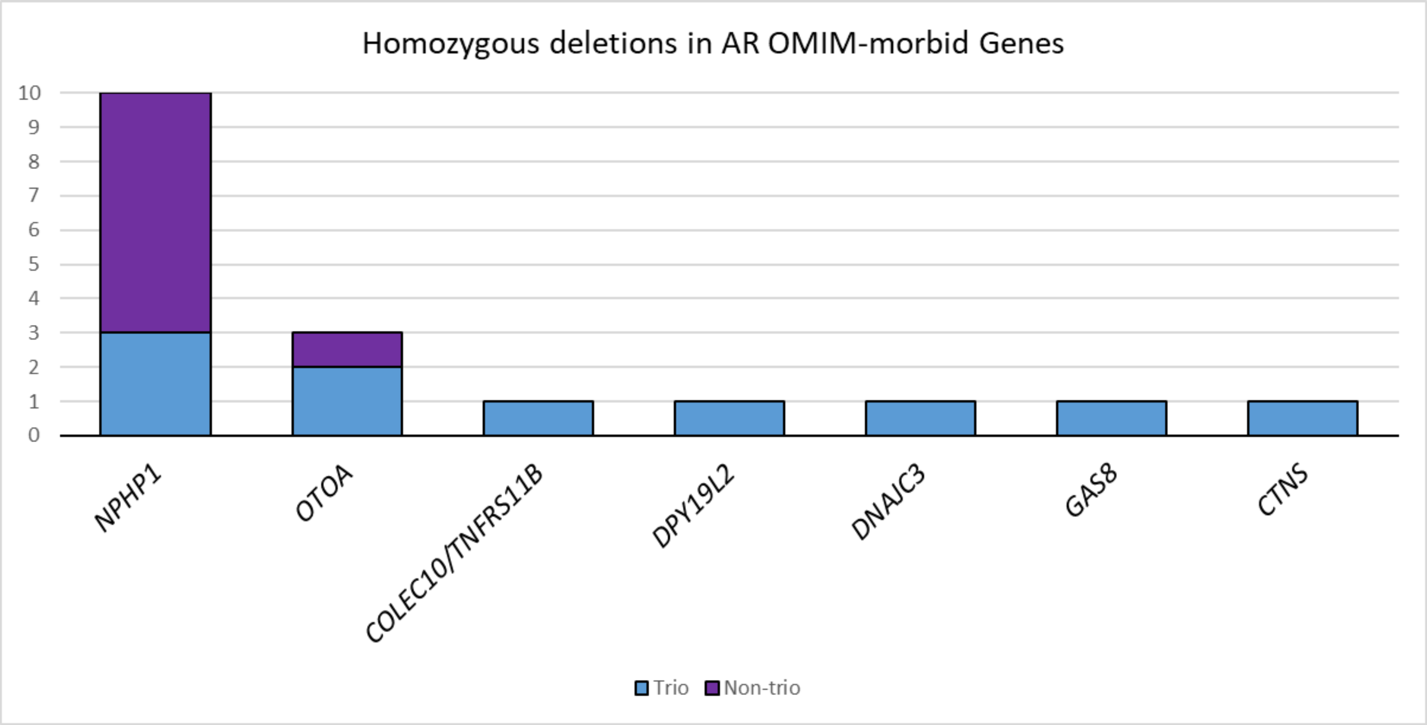

Figure S8. Total number of parent-child trio and non-trio patients with corresponding gene deletions

Bar chart showing total number of parent-child trio and non-trio patients solved for corresponding gene deletions.

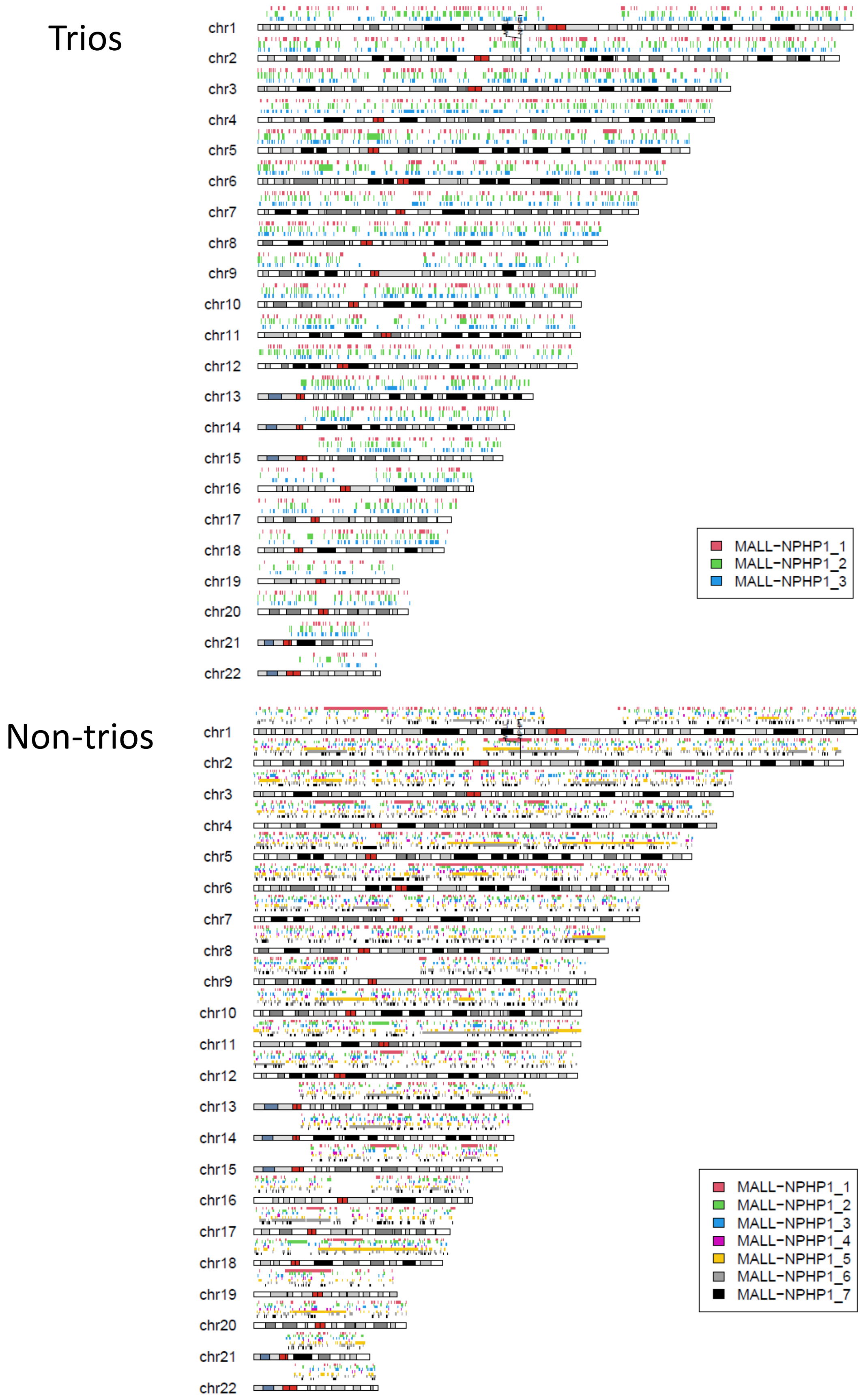

Figure S9. Autozygosity plots for parent-child trios and non trio patients with homozygous deletions involving *NPHP1*

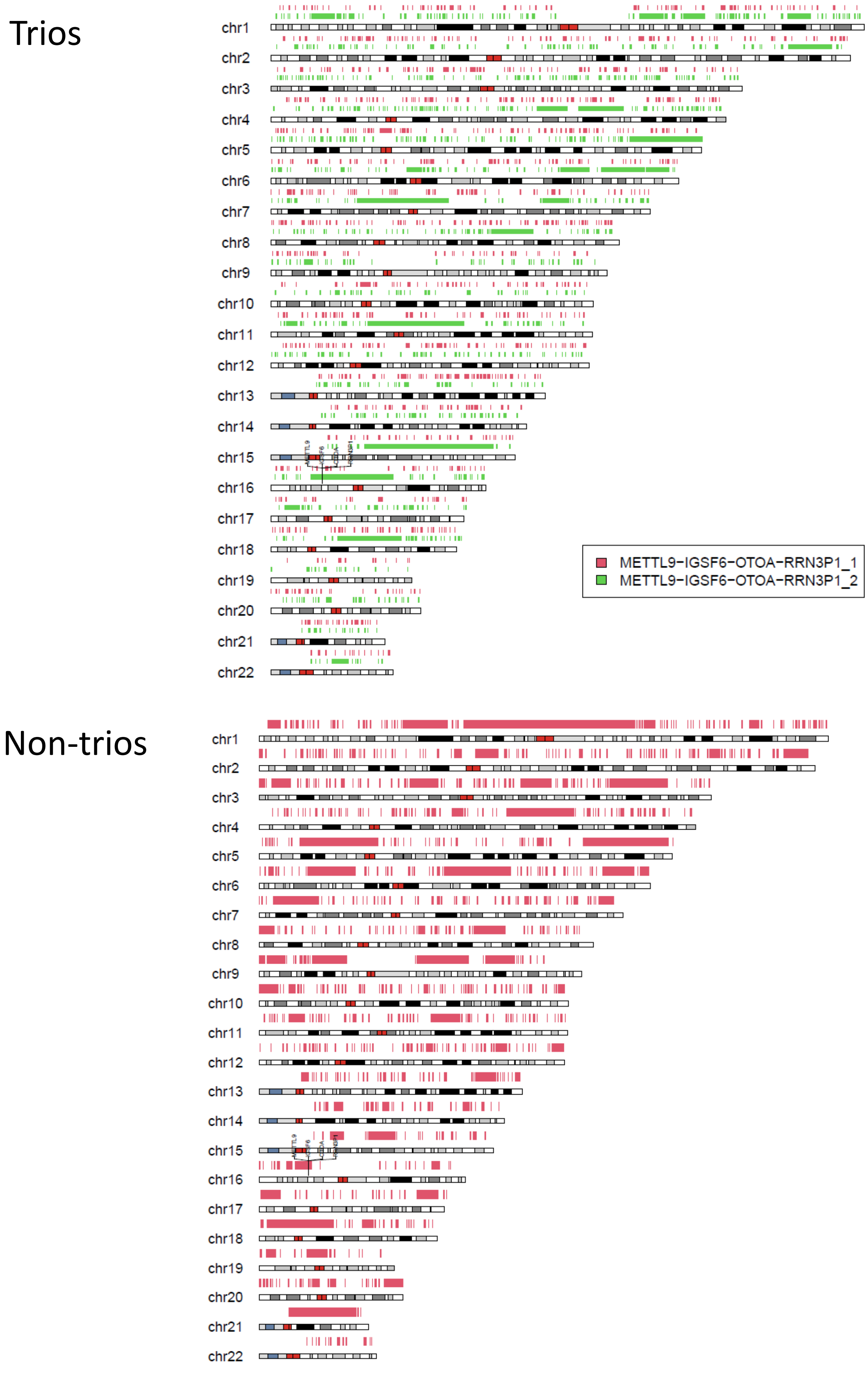

Figure S10. Autozygosity plots for parent-child trios and non trio patients with homozygous deletions involving *OTOA*

**1000G Population**	***NPHP1*** **cn1**	**cn2**	**cn3**	**cn4**
African Ancestry in Southwest US	1	59	1	0
African Caribbean in Barbados	1	93	2	0
Bengali in Bangladesh	1	98	2	0
British in England and Scotland	3	88	0	0
Chinese Dai in Xishuangbanna, China	0	88	5	0
Colombian in Medellin, Colombia	0	97	0	0
Esan in Nigeria	0	106	0	0
Finnish in Finland	0	98	1	0
Gambian in Western Division, The Gambia - Mandinka	0	120	0	0
Gujarati Indians in Houston, TX	0	103	0	0
Han Chinese in Beijing, China	2	99	2	0
Han Chinese South	2	110	0	0
Iberian populations in Spain	0	107	0	0
Indian Telugu in the UK	0	99	4	1
Japanese in Tokyo, Japan	0	102	2	0
Kinh in Ho Chi Minh City, Vietnam	0	98	3	0
Luhya in Webuye, Kenya	0	98	1	0
Mende in Sierra Leone	1	87	0	0
Mexican Ancestry in Los Angeles, California	0	65	0	0
Peruvian in Lima, Peru	1	86	0	0
Puerto Rican in Puerto Rico	2	101	1	0
Punjabi in Lahore, Pakistan	0	103	1	0
Sri Lankan Tamil in the UK	0	101	3	0
Toscani in Italy	0	105	2	0
Utah residents (CEPH) with Northern and Western European ancestry	0	122	0	0
Yoruba in Ibadan, Nigeria	0	122	0	0
**TOTAL**	**14**	**2555**	**30**	**1**

1000 Genomes
A Deep Catalog of Human Genetic Variation

Genomics England
Cancer cohort

14 cn1 out of 2600 individuals
NPHP1 prevalence = 0.0054

NPHP1 cn1 prevalence = 0.0035

Figure S11. *NPHP1* copy number frequency in 1000 Genomes dataset using SYNOD CNV calling pipeline

cn1, cn2, cn3 and cn4 denotes copy number 1, 2, 3 and 4, respectively.

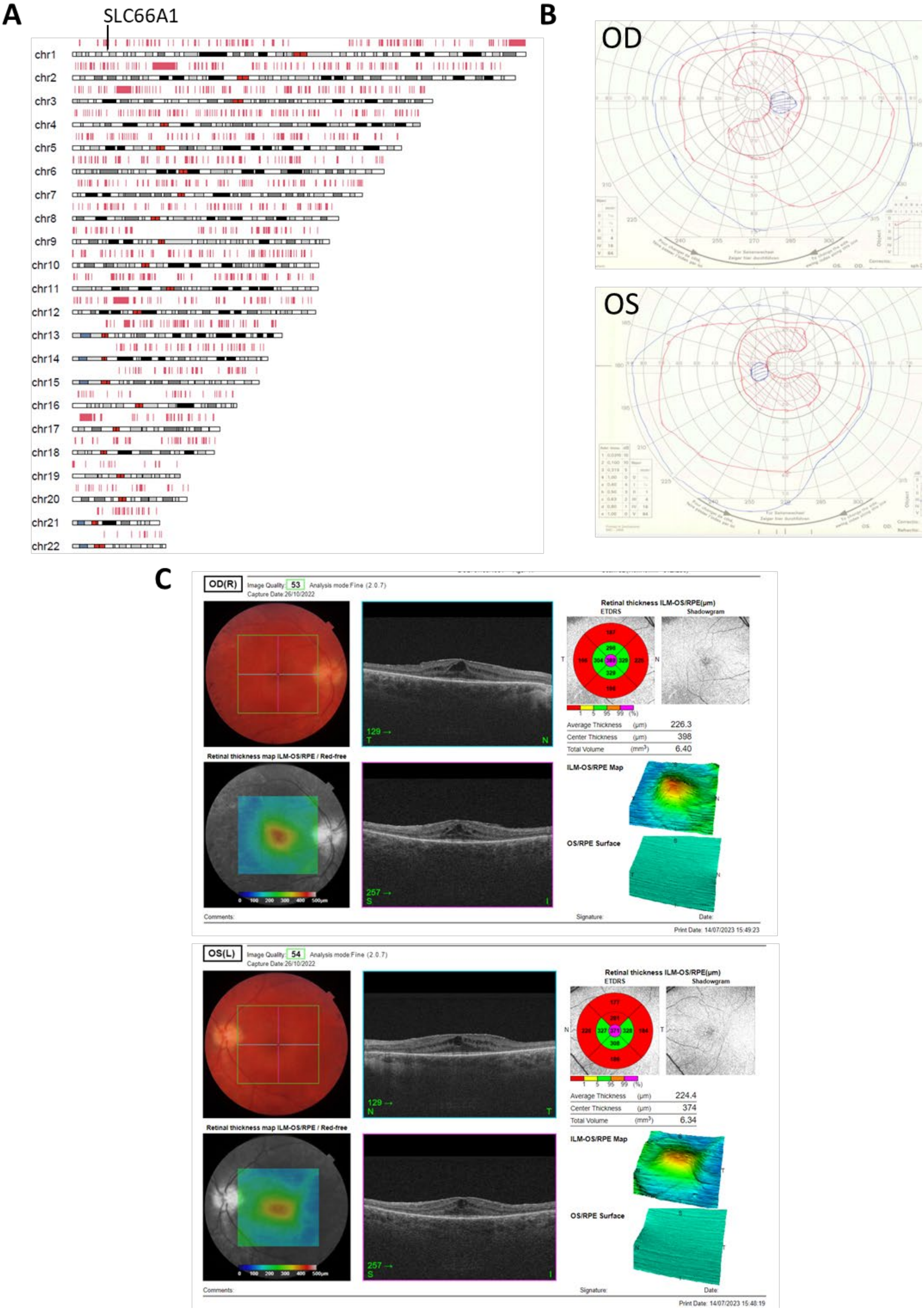
A
SLC66A1
chr1
chr2
chr3
chr4
chr5
chr6
chr7
chr8
chr9
chr10
chr11
chr12
chr13
chr14
chr15
chr16
chr17
chr18
chr19
chr20
chr21
chr22
B
OD
OS
C
OD(R)
Image Quality: 53
Analysis mode:Fine (2.0.7)
Capture Date:26/10/2022
Retinal thickness ILM-OS/RPE(μm)
ETDRS
Shadowgram
Average Thickness (μm) 226.3
Center Thickness (μm) 398
Total Volume (mm3) 6.40
Retinal thickness map ILM-OS/RPE / Red-free
ILM-OS/RPE Map
OS/RPE Surface
Comments:
Signature:
Date:
Print Date: 14/07/2023 15:49:23
OS(L)
Image Quality: 54
Analysis mode:Fine (2.0.7)
Capture Date:26/10/2022
Retinal thickness ILM-OS/RPE(μm)
ETDRS
Shadowgram
Average Thickness (μm) 224.4
Center Thickness (μm) 374
Total Volume (mm3) 6.34
Retinal thickness map ILM-OS/RPE / Red-free
ILM-OS/RPE Map
OS/RPE Surface
Comments:
Signature:
Date:
Print Date: 14/07/2023 15:48:19

Figure S12. Autozygosity plot and ophthalmology phenotype for individual II.1 (Fig 2B) carrying a homozygous *SLC66A1* deletion

A. Autozygosity plot for patient II:1 with homozygous deletion involving *SLC66A1.*

B. Goldmann manual perimetry showing bilateral midperipheral visual field loss sparing central 15 degrees and outer peripheral visual field.

C. Topcon imaging report for right (OD) and left (OS) eye, showing colour fundal images (top left), black & white fundal images (bottom left), cross-sectional optical coherence tomography (OCT) imaging of macula (middle panels), total retinal thickness measurements (top right), and thickness maps of whole retina and photoreceptor-retinal pigment epithelial (RPE) interface (bottom right).

A

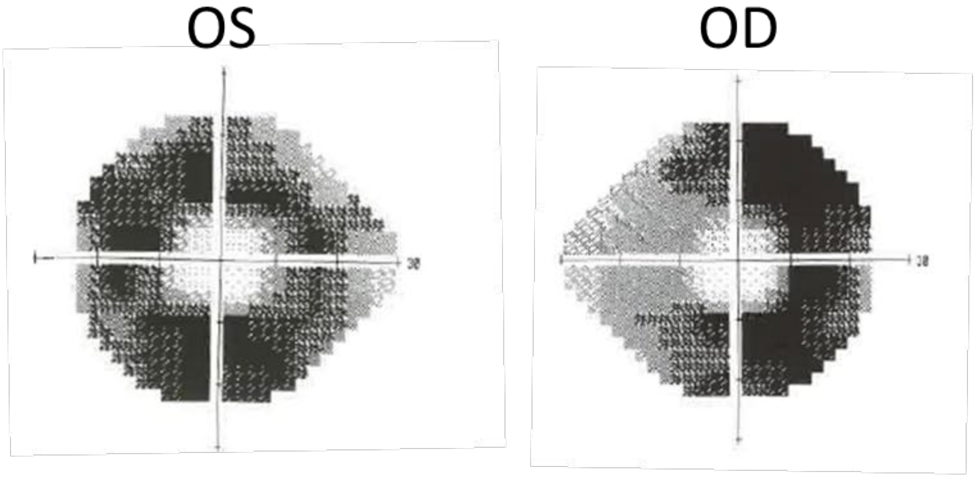

B

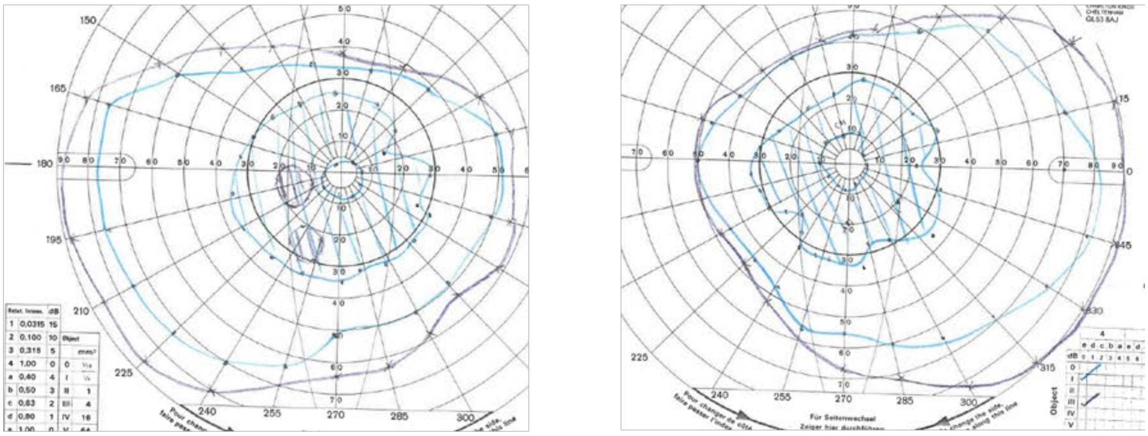

C

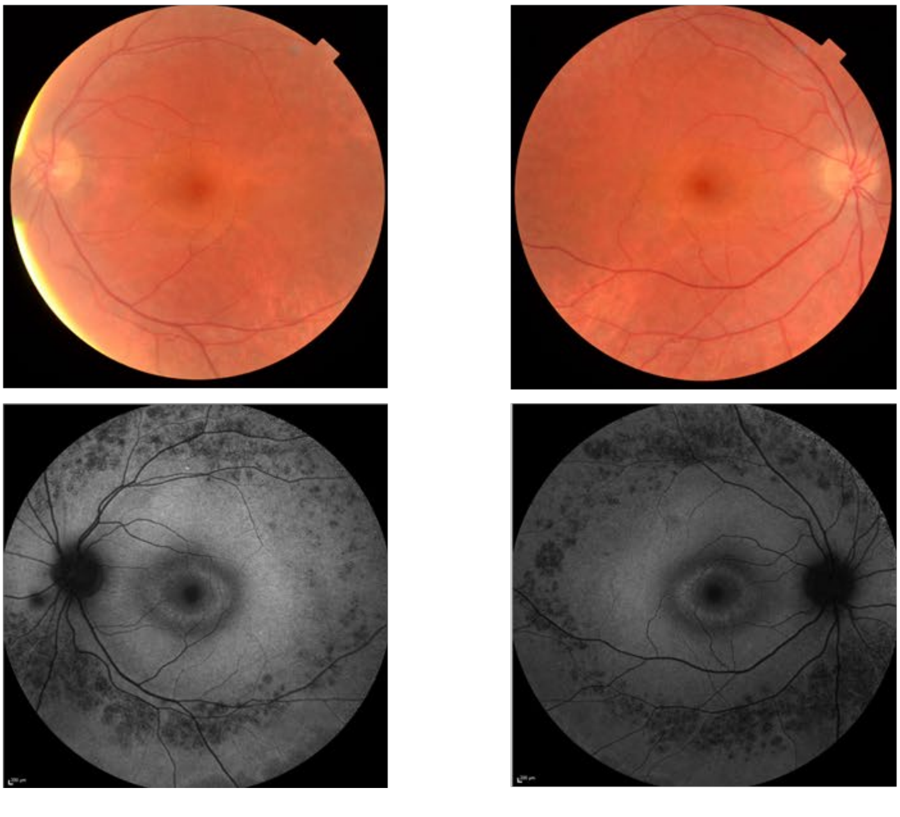

Figure S13. Retinal phenotype for individual II.2 (Fig 2B) carrying a homozygous *SLC66A1* deletion

Right (OD) and Left (OS) visual field loss sparing central 5-10 degrees and outer peripheral visual field, demonstrated by (A) Humphrey 24:2 automated perimetry, and (B) Goldmann manual perimetry. (C) Bull's eye maculopathy and bone spicule pigmentation following vascular arcades (top panels are colour photos; bottom panels are black & white autofluorescence images).

A

	Normal Mean	Normal Range	Left eye	Right eye
**RETINAL RESPONSES**				
**Max response (white, dark adapted)**				
a wave latency (msec)	13.4	10.0 to 15.9	NR	NR
a wave amplitude (uV)	-18.9	-11.1 to -26.6	NR	NR
b wave latency (msec)	61.0	46.1 to 75.9	NR	NR
b wave amplitude (uV)	65.9	31.1 to 110.7	NR	NR
**Rod response**				
Amplitude (uV)	58.3	34.7 to 81.9	NR	NR
**Cone response**				
a wave latency (msec)	13.4	11.5 to 15.4	NR	NR
a wave amplitude (uV)	-6.3	-4.3 to -8.5	NR	NR
b wave latency (msec)	33.0	29.2 to 36.7	NR	NR
b wave amplitude (uV)	10.4	2.9 to 18.0	NR	NR
**30 Hz flicker**				
Implicit time (i) (msec)	28.6	25.5 to 31.8	30.4	31.0
Implicit time (ii) (msec)	61.7	58.4 to 64.9	62.0	62.2
Implicit time (iii) (msec)	94.8	91.5 to 98.1	99.0	96.6
Mean amplitude (uV)	14.4	9.2 to 19.7	2.5	3.1

	Normal Mean	Normal Range	Binocular	
**CORTICAL RESPONSES**				
**Flash VEP**				
P1 latency (msec)	74.4	50.4 to 98.4	95.0	
P1 amplitude (uV)	16.2	4.0 to 28.4	4.3	
N1 latency (msec)	124.9	92.2 to 157.6	128	
N1 amplitude (uV)	-8.6	-20.3 to 3.1	1.5	
P2 latency (msec)	175.6	144.8 to 206.4	190.0	
P2 amplitude (uV)	8.0	-5.8 to 21.8	3.8	
**30 Hz flicker**				
Latency (i) (msec)	16.3	8.4 to 24.2	21.4	
Latency (ii) (msec)	47.9	38.7 to 57.1	53.0	
Latency (iii) (msec)	81.0	74.3 to 87.7	83.0	
Mean amplitude (uV)	6.7	3.0 to 10.4	4.2	
**PR-VEP**			Left eye	Right eye
Full field P100 latency (msec)	100.2	90.7 to 109.7	107.5	105.5
Right half field P100 latency (msec)	100.6	89.4 to 111.8	106.0	100.0
Left half field P100 latency (msec)	100.6	89.4 to 111.8	108.5	108.0

B

	Normal Median	Normal Range	Left eye	Right eye
**RETINAL RESPONSES**				
**Dark adapted retinal responses**				
**Rod**				
b wave latency (msec)	63.0	45.6 to 94.0	115	110
b wave amplitude (uV)	254.4	143.8 to 511.5	81.1	70.4
**High-intensity**				
a wave latency (msec)	12.5	11.0 to 15.0	28	27
a wave amplitude (uV)	-214.4	-72.0 to -353.3	-149.3	-139.3
**Maximal (combined response)**				
a wave latency (msec)	16.0	14.7 to 23.0	31	30
a wave amplitude (uV)	-169.8	-54.3 to -253.1	-125.8	-125.7
b wave latency (msec)	46.0	32.6 to 53.0	49	48
b wave amplitude (uV)	353.4	141.6 to 620.5	112.9	94.0
**Oscillatory potentials**				
Summed amplitude (uV)	158.5	41.4 to 229.9	24.3	22.9
**Light adapted retinal responses**				
**Cone**				
a wave latency (msec)	14.0	13.0 to 16.3	18/28	16/29
a wave amplitude (uV)	-30.9	-8.5 to -50.1	-21.7	-16.9
b wave latency (msec)	29.0	28 to 34.3	35	35
b wave amplitude (uV)	141.6	33.2 to 235.9	26.0	27.0
**30Hz flicker**				
Implicit time 1	26.5	24.0 to 30.3	32	32
Implicit time 2	59.5	57.0 to 64.0	65	65
Implicit time 3	93.0	90.0 to 97.7	97	97
Implicit time 4	125.5	123.0 to 143.0	131	130
Implicit time 5	159.0	156.0 to 169.2	164	164
Mean amplitude	87.7	23.4 to 175.7	27.0	31.4

Figure S14. Electrodiagnostic testing in siblings with *SLC66A1* homozygous gene deletions

Electrodiagnostic testing in siblings II:1 (A) and II:2 (B) is consistent with rod-cone dystrophy.

A

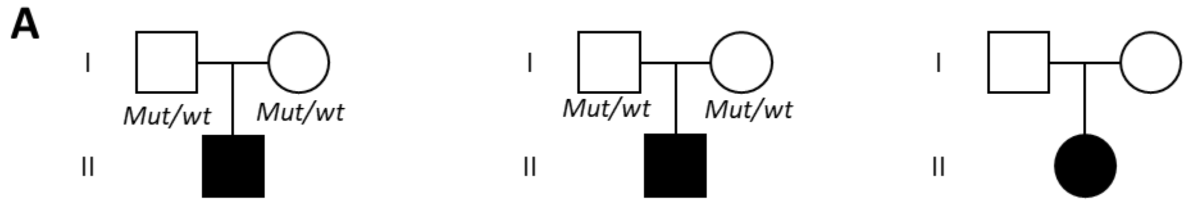

Early-onset severe rod-cone dystrophy
Narrow and long thorax
Polyarticular arthritis
Radial bowing
Abnormality of distal humeral epiphysis
Moderate conductive hearing impairment

Rod-cone dystrophy
Progressive visual loss
Not solved in GEL – no TIER1 or TIER2

Rod-cone dystrophy
Progressive visual loss
Thyroid medullary carcinoma
Endometriosis
Leiomyoma of uterus
Type 2 diabetes mellitus
Not solved in GEL – no TIER1 or TIER2

ID	Gene	Genomic position (GRCh38)	REF	ALT	GT	Nucleotide change	Amino acid change	dbSNP	gnomAD
SLC66A1-1 15-19y	*SLC66A1*	1:19327258	ACT	A	1/1	c.655_656del	p.(Leu219ValfsTer67)	/	n.d.
	SPATA7 **(TIER1)**	**14:88416725**	**C**	**T**	**1/1**	**c.253C>T**	**p.(Arg85Ter)**	**rs140287375**	**18/250,440/0**
	IHH (TIER2)	2:219055586	G	A	1/1	c.857C>T	p.(Pro286Leu)	rs140093604	278/249,344/2
SLC66A1-2 40-44y	*SLC66A1*	1:19326293	GCGCCACAC	G	1/1	c.435_442del	p.(Thr146AlafsTer11)	rs758581408	1/245,576/0
SLC66A1-3 65-69y	*SLC66A1*	1:19326293	GCGCCACAC	G	1/1	c.435_442del	p.(Thr146AlafsTer11)	rs758581408	1/245,576/0

B

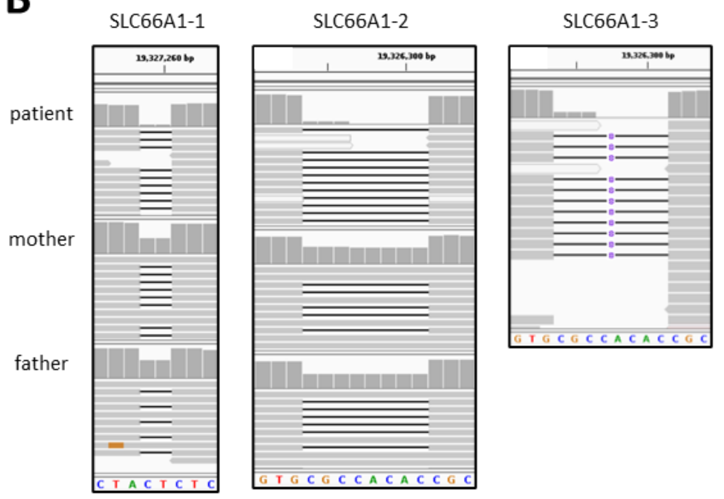

C

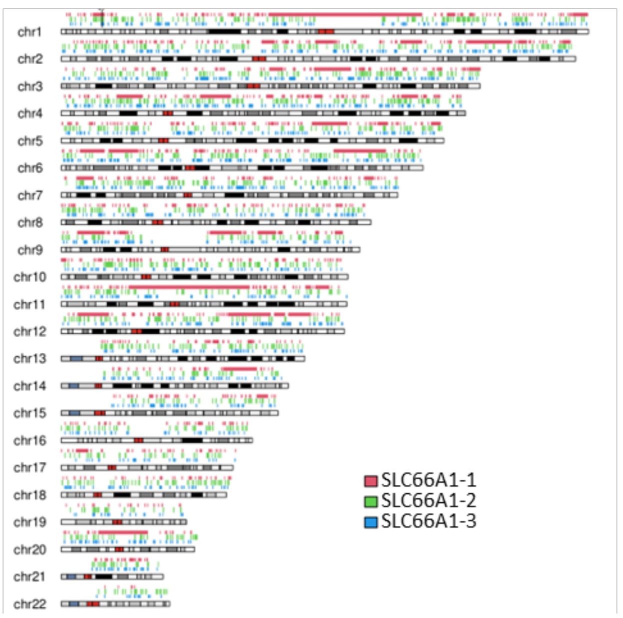

Figure S15. Identification of additional patients with *SLC66A1* variants and retinal phenotypes

A. Pedigree diagram and clinical phenotypes of 3 patients (SLC66A1-1, SLC66A1-2 SLC66A1-3) within the GE100kGP rare disease dataset with *SLC66A1* homozygous loss of function variants. Details of genetic variants and additional findings within tiering datasets are shown. (NM_001040125.2 for *SLC66A1*, NM_018418.5 for *SPATA7*, NM_002181.4 for *IHH*)

B. IGV visualisation of BAM files to show *SLC66A1* alleles for patients (SLC66A1-1, SLC66A1-2, SLC66A1-3)

C. Autozygosity plots for the three individuals (SLC66A1-1, SLC66A1-2, SLC66A1-3).
